# Whole-genome resequencing of tea grey geometrid provides insights into their population structure and adaptation to tea crops

**DOI:** 10.1007/s44297-024-00026-z

**Published:** 2024-06-01

**Authors:** Ruizhong Yuan, Yusi Chen, Xiaogui Zhou, Xiaohan Shu, Zhaohe Lu, Pu Tang, Xiqian Ye, Zhizhi Wang

**Affiliations:** 1 Institute of Insect Sciences, College of Agriculture and Biotechnology, Zhejiang University, Hangzhou, 310058 China; 2 State Key Lab of Rice Biology, Ministry of Agriculture Key Lab of Molecular Biology of Crop Pathogens and Insects, and Zhejiang Provincial Key Laboratory of Biology of Crop Pathogens and Insects, Zhejiang University, Hangzhou, 310058 China; 3 Hainan Institute, Zhejiang University, Sanya, 572025 China; 4 Key Laboratory of Tea Biology and Resources Utilization, Ministry of Agriculture, National Center for Tea Improvement, Tea Research Institute, Chinese Academy of Agricultural Sciences, Hangzhou, 310008 China; 5 Guangdong Laboratory for Lingnan Modern Agriculture, Guangzhou, 510642 China

**Keywords:** Tea grey geometrid, Genomic variation, Population genetics, Local adaptation, Evolution history

## Abstract

Tea grey geometrid, *Ectropis grisescens* (Lepidoptera: Geometridae), is a chewing defoliator of high fecundity and gluttony, whose larvae heavily consume leaves and shoots, and consequently cause significant losses to tea crops in terms of both yield and quality. Nevertheless, a lack of population genome information has hindered previous research on the species as a tea crop pest. Here, we re-sequenced genomes of 43 tea grey geometrid individuals representing 13 distinct geographic regions and 12 individuals of its sibling species, the tea geometrid *Ectropis obliqua* (Lepidoptera: Geometridae), collected from four different sites in China. A genomic variation atlas of 627,569 single-nucleotide polymorphisms (SNPs) from whole-genome resequencing of 43 individuals was identified. Surprisingly, the populations of the tea grey geometrid (EG) are found to be assigned into two distinct subpopulations, EGA and EGB. The sample locations indicate that the subpopulation EGA is mainly distributed in the region of Zhejiang Province, where it co-occurred with tea geometrids (EO). The EGB subpopulation is distributed in the southern and central areas of China. Demographic history analysis has revealed fluctuations in population size of the tea grey geometrid over time, but following tea cultivation, the population size has started to stabilize. Meanwhile, the migration event analysis has uncovered a significant migration event between EGA and EO. Furthermore, genes (such as *P-glycoprotein* and *lactase*) under selection show possible metabolic adaptation of tea grey geometrid to the tea plants. These findings provide a broader foundation and additional resources for understanding the geometrid evolution and the genetic mechanisms underlying agriculturally important traits.

**Supplementary Information:**

The online version contains supplementary material available at 10.1007/s44297-024-00026-z.

## Introduction

The tea, *Camellia sinensis* (Ericales: Theaceae), is a global economic crop [1–4], whose annual plantation acreage reached 3.31 million hectares in China, in 2022 (https://www.statista.com). As an integral component of traditional Chinese products, tea holds a significant position in the international production and trade market of China [5]. China is one of the birthplaces of the tea crop and has a history of thousands of years of tea cultivation. During the long-term evolutionary process, tea crops radiate and spread from the place of origin and are widely cultivated in more than 60 countries around the world through continuous artificial introduction and cultivation [6]. The domestication and selection of tea have resulted in different levels of genetic diversity and population differential, alongside the diversity level of morphological, physiological and biochemical characteristics, and sometimes biotic or abiotic stress resistance [7–9]. With many beneficial effects on human health, tea is prepared in a simple process and can be directly brewed for drinking [10, 11]. Therefore, tea products require extremely high standards of quality. At the same time, a number of variables, including pest infestation and its management, have a direct impact on the quantity and quality of tea products.

In China, tea grey geometrid, *Ectropis grisescens* (Lepidopotera: Geometridae) (EG), is the most important phyllophagous pest of tea crop [5], and seriously affects the yield and quality of tea crops and causes great economic losses to the tea production [12–19]. In addition, the tea geometrid, *Ectropis obliqua* (Lepidopotera: Geometridae) (EO), is a sibling species to the tea grey geometrid. They share a similar appearance and cause similar symptoms in tea crops [5, 20–23], but differ in geographic distribution- tea grey geometrid is extensively dispersed throughout major tea-producing areas of China while tea geometrid is primarily found near around Taihu Lake [14, 24]. The biological characteristics of the two sibling species also differ, including reproductive capacity, tolerance to virus infections, endosymbiont (*Wolbachia*) composition and virulence composition [25–30]. Besides, the interaction between geometrids and tea crops leads to reciprocal evolutionary change. For instance, the volatiles produced when tea grey geometrid larvae feed can stimulate neighbouring tea crops to emit beta-Ocimene, enhancing the ability of healthy neighbouring plants to repel adult tea grey geometrids [31]. On the other hand, the feeding of tea grey geometrid larvae induces a series of metabolic variations including flavonoids (catechin, quercetin, epigallocatechin gallate, etc.), resulting in a direct defense reaction, which is regulated by signaling pathways involving jasmonic acid, ethylene, and auxin [32, 33]. The cultivation of tea varieties in China is mainly divided into two tea varieties, *Camellia sinensis* var. *sinensis* and *C. sinensis* var. *assamica*, which provide a good model for host-driven pest evolution. Besides, it is an essential element of integrated pest management that recognises the differences between different geographic populations of tea grey geometrid [34]. However, larger-scale genetic information analysis is still lacking.

In this study, we collected samples from major tea-growing regions in China where the tea grey geometrid has recently caused severe economic losses due to outbreaks. To elucidate the molecular mechanism behind the frequent outbreaks of *E. grisescens*, we employed next-generation sequencing technology to sequence the genomic data of the tea grey geometrid by analyzing the genetic diversity and population differentiation. We observed two distinct subpopulations (EGA and EGB) in the natural geographic distribution of the tea grey geometrid, with EGA mainly distributed in the region of Zhejiang Province (co-occurring with tea geometrids). Furthermore, we constructed a population genetic map and identified genes (such as *P-glycoprotein* and *lactase*) under selection that are possibly associated with tea grey geometrid’s adaptation to tea crops. These results shed light on the evolution and genetic mechanisms of the tea grey geometrid, revealing important traits and interactions with tea crops. The genomic variation atlas produced in this study represent a comprehensive resource for future research on tea grey geometrid and related species.

## Results

### Population sampling and sequencing

**Fig. 1:**
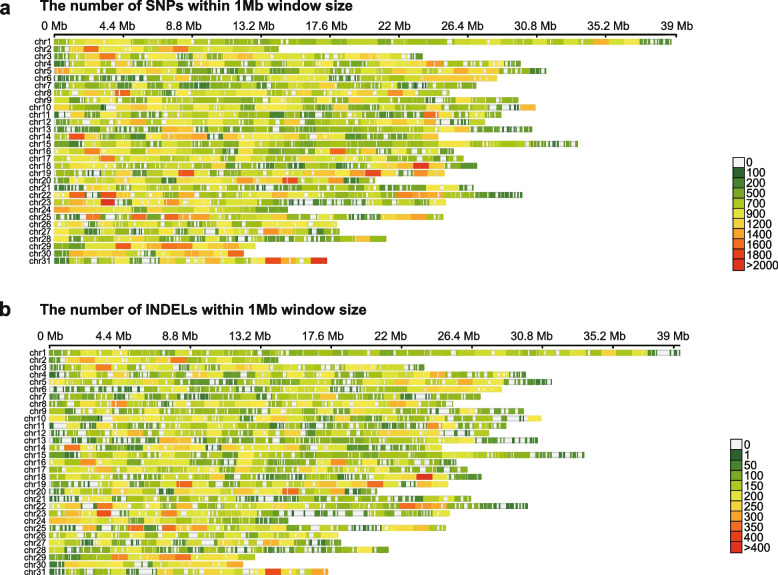
The density of SNPs/InDels of tea grey geometrid. **a** The density of SNPs in 31 chromosomes of tea grey geometrid. **b** The density of InDels of 31 chromosomes of tea grey geometrid

A total of 43 tea grey geometrid individuals from 13 major geographic distribution areas were selected for whole genome resequencing, and the sample set covers a large part of the tea-growing region of China (Table S1) [14, 23]. Twelve tea geometrid samples were also sequenced as the outgroup [5, 20–23]. In total, we generated 3.31 Tb of data (11.03 billion read pairs) and mapped these data to the reference genome [13], resulting in an average sequencing depth of 34.75 × . Based on the mapping results, we ultimately identified 6.27 million genetic variants in tea grey geometrid, including 627,569 high-quality single nucleotide polymorphisms (SNPs) and 131,054 small insertions and deletions (InDels). Our analysis revealed a global SNP density of one genetic variant per 1,013 bp on average. Interestingly, the intron regions accounted for 83.03% of the SNPs, while the exon region represented only 3.33% of the SNPs. Within the exon region, a total of 6,351 non-synonymous SNPs and 18,695 synonymous SNPs were identified. SNP distribution was observed across various chromosomes in the genome, and high-density SNP regions were identified in Chr14, Chr18, Chr19, Chr23, and Chr31 (Fig. 1a). In addition, InDels density showed a similar trend as SNP density, with the highest density region presented in Chr18, suggesting comparable evolutionary and selection pressures on these genome fragments (Fig. 1b). In summary, we observed a relatively high level of whole-genome diversity within the tea grey geometrid population, indicating a high effective population size, thus creating a large genetic pool that may be useful in the future for biological control purposes [35].

### Population structure and phylogenetic analysis of tea grey geometrid

**Fig. 2:**
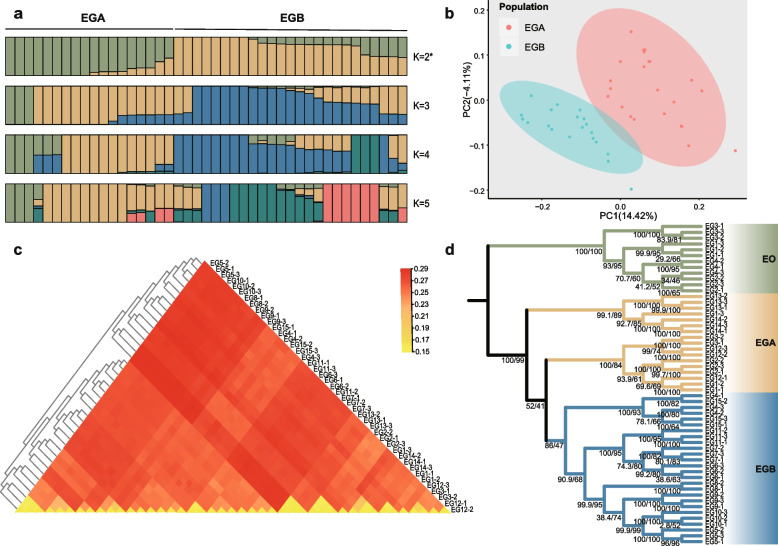
Population structure of tea grey geometrid. **a** Admixture plot that indicates population structures for K from 2 to 5. “*” means that K = 2 is the optimal K value. **b** PCA plot analysis of tea grey geometrid samples. The confidence interval was set to 95%. **c** IBS genetic distance of tea grey geometrid samples. **d** The phylogenetic analysis of all the samples using SNP data. The phylogenetic was constructed by the maximum likelihood (ML) method and SNP data of tea geometrid samples was used as outgroup (EO). The bootstrap/alert values are indicated on the node

To reveal the natural population relationships among various geographical populations of tea grey geometrid, we utilized the SNP dataset for further analysis. In general, population structure analysis (Fig. 2a, Fig. S1, Table S2), principal component analysis (PCA) (Fig. 2b, Table S3) and identity by state (IBS) genetic distance (Fig. 2c, Table S4) supported the divergent of the tea grey geometrid samples. Samples from Zhejiang Province and Henan Province were assigned EGA (*n* = 17), while individuals from other regions were named EGB (*n* = 26). Phylogenetic analysis also showed all the tea grey geometrid samples were divided into two clusters (Fig. 2d). These results suggest that the populations of tea grey geometrid in the primary tea regions of China have differential into two subpopulations, one of which is mainly distributed in Zhejiang Province, while the other is evenly distributed throughout other areas (Table S1). Obviously, the population diversification would be a crucial factor to cause a higher genetic variation in tea grey geometrid.

### Demographic history and migration event analysis

**Fig. 3:**
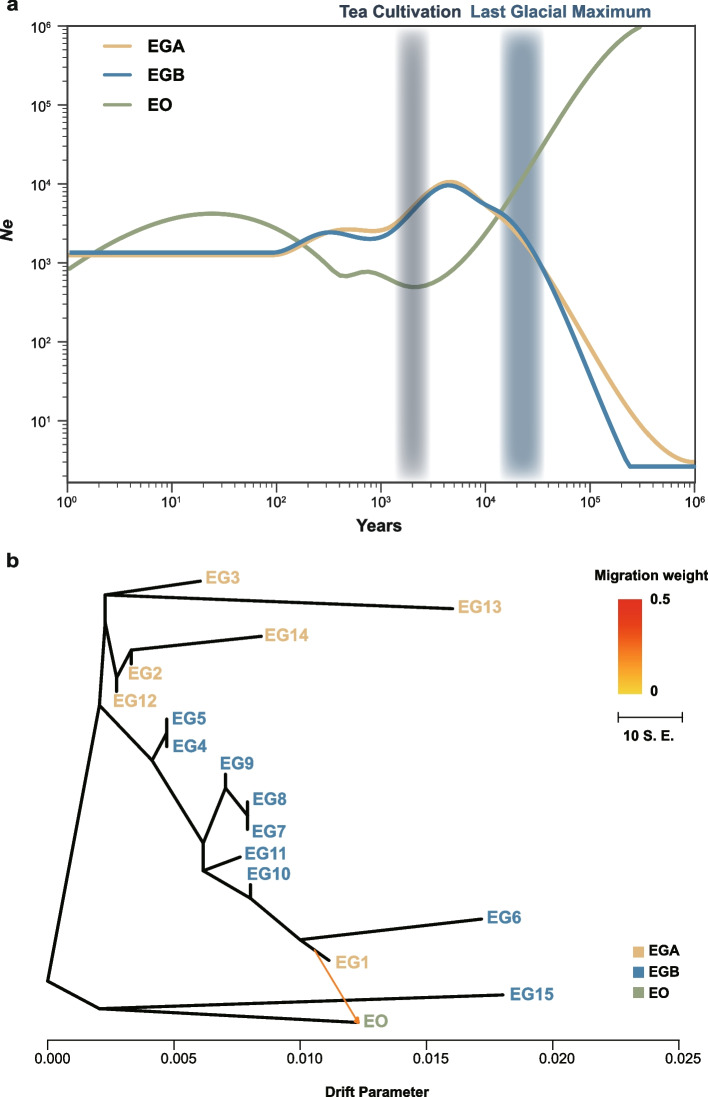
Demographic history and migration event analysis. **a** The demographic analysis of tea grey geometrid and tea geometrid. Tea cultivation period and last glacial maximum period were marked as bar in plot with slight blue and weight blue, respectively. **b** The migration event in EGA, EGB and EO. Arrow means migration event orientation in different subpopulation and population. The color range of arrows means standard error (S. E.) value of migration weight

To investigate the factors leading to the divergence among the geometrid wild lineage (EGA, EGB and EO), we generated historical effective population size change curves (Fig. 3a). The demographic history of tea grey geometrid suggested a fluctuation in the estimated effective population size (*N*_*e*_), characterized by a sequential contraction and expansion over time. After the last glacial period, the population sizes of EGA and EGB remained relatively stable, fluctuating between approximately 1,000 and 10,000. However, following the advent of tea cultivation [36], the effective population size of both subpopulations began to level off. During recent evolution, EGB has shown a similar diminutive effective population size to EGA. Genetic drift emerging within small subpopulations has been a potential contributing factor in the natural evolution of these populations. In addition, the population size change of EO exhibited a contrasting trend to that of EGA and EGB. In contrast, EO demonstrated persistent fluctuations since the last glacial period, suggesting that the effective population size of EO is vulnerable to external factors.

To explore possible genetic exchange, we performed a TreeMix analysis to infer population differentiation and mix model. The optimum value of the migration edge (m) ranging from 1 to 10 was evaluated. When delta m value reached the smallest, the optimal m was determined to be 1. Further, the maximum likelihood tree and reside heat map were conducted with m = 1 (Fig. S2-3). This value points to a significant migration event among EGA, EGB and EO. Interestingly, the migration event occurs between the population EG1 and EO, of which the former was found in Hangzhou, Zhejiang Province, and the latter was distributed around Taihu Lake (Fig. 3b) [14, 24]. The genetic drift parameter between EG1 and EO was found to be less than 0.002, indicating a resemblance in genetic structure between these two populations.

### Selection signal as a potential force for forming subpopulation

**Fig. 4:**
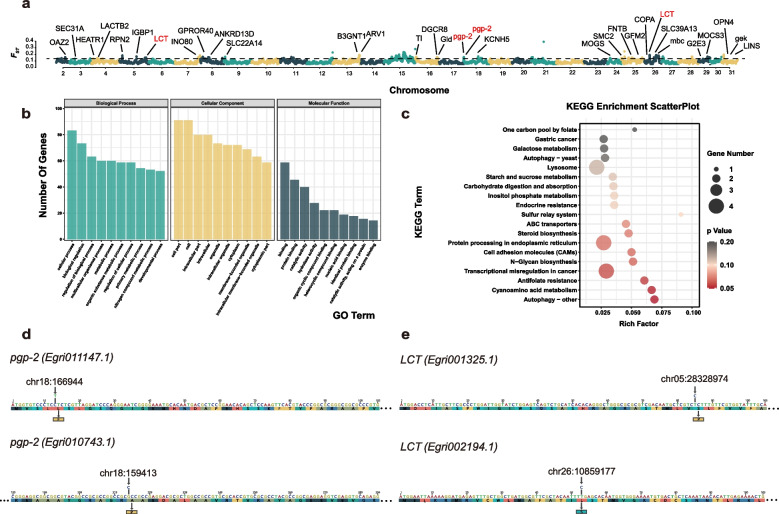
Sweep selection of SNPs in the tea grey geometrid. **a** *F*_*ST*_ values. Window = 5000 kb, 5% as candidate genes, and > 0.25 as key windows that influence relationship between each subpopulation. **b** GO enrichment of 222 candidate genes. **c** KEGG pathway enrichment of 222 candidate genes. **d** The SNP in two *pgp-2* genes. **e**. The SNP in two *LCT* genes

We performed selection signal detection in all the EG samples based on population differentiation monitoring and locus frequency monitoring. The nucleotide polymorphism indices (*θ*_*π*_) of the two subpopulations showed some differences (Table S5), proportional to the genotypic diversity within a single population, with EGA and EGB of the wild line being closer. The inter-population genetic differentiation index (*F*_ST_) was calculated to obtain *F*_ST_ values [37], and the *F*_ST_ value between 0.25 and 1 for a single genetic window indicates a high genetic differentiation between populations (Fig. 4a, Table S6). Chr15 contains the highest number of SNP sites in the top 5% of the *F*_*ST*_ windows, while no SNP was found in exon regions. We computed the genetic diversity (*θ*_*π*_) and Tajima's *D* value (Table S7) on Chr15. According to the results of the *π* value shown in Fig. S4, we observed that regions near the both ends of chromosome 15 (the region 0 ~ 15 Mb and the region 25 ~ 35 Mb) exhibited a higher value of *π*, indicating increased genetic diversity towards the chromosome ends. As for Tajima's *D* value (Fig. S5), we observed that the majority of sites on chromosome 15 display positive values, indicating a higher frequency of intermediate mutations in the population. This could be attributed to influences such as population expansion, positive selection, or rare variants.

A selection of 5% *F*_ST_ windows yielded 222 genes (Table S8) and a total of 348 SNPs were found in their exon region. To understand the function of the selected genes, GO enrichment and KEGG pathway were performed. Functional annotation of these 222 candidate genes was conducted by EggNOG [38] (Table S8). Generally, the GO term of these genes mainly focused on the cellular process, biological regulation, regulation of biological process, etc. (Fig. 4b, Fig. S6), while the KEGG pathway mainly focused on transcriptional regulation, protein processing and modification, etc. (Fig. 4c). Of note, several exon-occurring nonsynonymous SNPs were found in genes belonging to ABC transporters and carbohydrate metabolism. These genes includes two P-glycoprotein genes (*pgp-2*: *Egri011147.1* and *Egri010743.1*) from ABC transporter family, and two lactase genes (*LCT*: *Egri001325.1* and *Egri002194.1*) from glycoside hydrolase superfamily, which functions are related to digestion and xenobiotic metabolism [39, 40], such as the metabolism of catechins in geometrid moths [41]. In *Egri011147.1*, an SNP (chr18:166944, C to T) on the 13th base of the open reading frame (ORF) causes the encoded amino acid to change from leucine (L) to phenylalanine (F), and an SNP (chr18:159413, G to C) on the 223rd base of *Egri010743.1* ORF makes the encoded amino acid alanine (A) change to proline (P) (Fig. 4d). In addition, one amino acid change from serine (S) to proline (P) due to an SNP (chr05:28328974, T to C) on the 85th base of the ORF of *Egri001325.1* and leucine (L) to serine (S) owing to an SNP (chr26:10859177, T to C) on the 53rd base of *Egri002194.1* ORF (Fig. 4e). Furthermore, paralogous genes of *pgp-2* and *LCT* were screened from the genome of the tea grey geometrid for natural selection analysis. Phylogenetic trees were constructed, and the dN/dS ratio was calculated (Fig. S7-8, Table S9-10). Within *pgp-2*, the dN/dS ratio was 0.292 at base 13 and 0.373 at base 223. Both ratios exceeded the overall average of 0.279. Regarding *LCT*, the dN/dS ratio was 0.408 at base 85 and base 53. Both ratios were higher than the mean value (0.297). These findings suggested a likelihood of natural selection occurring at these loci. Thus, we propose that the diversification of tea grey geometrid subsets might be influenced by the interplay between tea geometrid and tea crops, mediated by the interaction of *pgp-2* and *LCT*.

## Discussion

In this study, the population structure, evolutionary patterns, and historical population changes of the wild lineage of the tea grey geometrid across different geographic populations were inferred. It was concluded that migration event at molecular level between tea grey geometrid and tea geometrid in the same geographic area played a significant role in subpopulation differentiation of wild lineage. The selective genes are likely associated with crucial physiological and biochemical processes that facilitate the insect’s adaptation to their ecological niche within the tea plantation ecosystem. This study illuminated genomic characteristics and interactions with tea crops that have contributed to evolution and genetic mechanisms of the tea grey geometrid.

Tea crops mainly grow in central and southern China [42]. Based on the results of this study, tea grey geometrid in central and southern China diverged into two subpopulations, namely EGA and EGB. As a result, EGA is mainly distributed in Zhejiang Province (EG1, EG2 and EG3), while EGB is found in other regions. It is worth noting that the tea grey geometrid population in Nanyang, Henan Province (EG3) belongs to the EGA and shares a genetic origin with the population in Zhejiang Province, suggesting that EG3 may have originated from Zhejiang Province and spread to the region through commercial activities or tea crop transportation. The EGB largely corresponded to the Jiangnan Tea Area and the South China Tea Area.

As *E*. *obliqua* co-occurred in tea crops, we added it to the demographic history and migration event analysis. After the last glacial maximum, both EGA and EGB populations showed smaller fluctuations in their effective populations, indicating that temperature was the main factor influencing their population size before the last glacial maximum. Once humans started tea cultivation, tea became an important economic crop, leading to tea-dominated agricultural ecosystems [36]. It is evident that following the domestication of tea crops, the effective population sizes of EGA and EGB have reached a stable state, closely associated with the emergence of tea-dominated agricultural ecosystems. On the other hand, the less distributed EO continued to fluctuate, suggesting that tea plants might also have an impact on the population size of these two species. This association is intricately connected to the widespread growth of tea cultivation and the relatively low proportion of wild tea crops. Besides, the resistance for different insecticides [43], development and reproduction [28, 29], and toxicity difference of microbiota [25–27] of the two sibling species also suggests EG is adapted well to domesticated tea crops. Consequently, the geographical distribution of tea grey geometrids is much larger compared to tea geometrids. However, the adaptability of EG and EO to the cultivation of tea needs further studies. Gene flow occurs between the subpopulation EGA and the sibling species EO. One possible factor is that EGA and EO are sympatric distributed in some areas which allows the sibling species to share similar habitats and therefore increase the likelihood of hybridization in the field [12], though it is demonstrated that the asymmetric mate of EG and EO produce sterile offspring. Corresponding to their hybrid results, the gene flow from EG to EO may have side effects on the EO population leading to narrower distribution.

We observed a relatively high level of whole-genome diversity within the tea grey geometrid genome population. The order Lepidoptera exhibits high genomic diversity [44–46], which may be a key reason for the significant number of SNPs occurring abundantly in coding regions. SNPs and InDels show a similar distribution pattern across chromosomes, with clustered occurrences on several chromosomes (Fig. 1b-c). This suggests that these chromosomal regions may harbor genes that contribute to intraspecific diversity and have great potential for exploration in the adaptation of tea grey geometrid to different tea cultivars.

From the enrichment analysis of GO and KEGG, it was observed that genes associated with subpopulation differentiation are predominantly found in metabolic pathways. The identified genes are primarily involved in the cellular process, biological regulation, regulation of biological process, etc. Additionally, the KEGG pathway analysis highlights transcriptional regulation, protein processing and modification, etc. The species of tea plants varied among samples, which are featured with diverse classes of metabolites [32, 47], which play an important role in the defense of tea plants against tea grey geometrid. For instance, the defense pathway involved in jasmonic acid and ethylene production in the tea plant is induced after the infestation of EG and catechin compounds accumulated, which, in turn, reduces the fitness of EG larvae [32]. Though EG can digest these metabolites, the activity of certain digestive enzyme, such as *LCT*, is inhibited by catechins [48]. Thus, the exon-occurring nonsynonymous SNPs in *LCT* possibly present an evolutionary way to adapt to domesticated tea crops. Collectively, the findings suggest that the selective genes are likely involved in important physiological and biochemical processes that facilitate the insect’s adaptation to its specific ecological niche within the tea plantation ecosystem.

The interaction between tea crops and tea grey geometrid is a natural phenomenon that involves considerations of adaptability and symbiosis. Of course, human activities can also profoundly affect the interaction between tea grey geometrid and tea crops, for example, long-term irrational use of chemical pesticides has led to an increase in resistance within the field populations of tea grey geometrids, further enhancing the likelihood of genetic variation occurring in these geometrids [49]. This could potentially have a significant impact on the yield and quality improvement of tea crops. Additionally, tea grey geometrids that escape during inspection and quarantine procedures may further spread within a larger geographical range through human activities. With a better understanding of the genetic adaptation of tea grey geometrid to tea crops, more effective strategies for pest management and crop protection can be developed, supporting sustainable agricultural practices [35].

## Materials and methods

### DNA sample preparation and sequencing

Tea grey geometrid samples were obtained from 13 different tea-producing areas and tea geometrid samples were obtained from four different tea-producing sites. All the samples were subjected to standard identification based on their distinct morphological characteristics and cytochrome c oxidase I (COI) barcodes information (Fig. S9). Subsequently, all the samples and were stored in 100% ethanol at -80 °C before genomic DNA extraction.

The genomic DNA was isolated using FastPure Cell/Tissue DNA Isolation Mini Kit (Vazyme Biotech Co., Ltd, Nanjing, China) according to the manufacturer’s instructions. The VAHTS Universal Plus DNA Library Prep Kit for Illumina (Vazyme Biotech Co., Ltd, Nanjing, China) was used for individual genomic DNA libraries using ~ 1 μg genomic DNA as input from each sample. The constructed libraries were sequenced on the Illumina NovaSeq sequencing platform (GrandOmics, Wuhan, China) and paired-end 150 bp reads were generated.

### Variation calling and annotation

Illumina paired-end sequenced raw reads for mapping were filtered using Fastp v0.20.1 [50] preprocessor to remove low-quality reads, adaptors, and reads containing poly-N using default parameters, and then clean reads were mapped to *E. grisescens* (ASM1756216v1, GCA_017562165.1) reference genome [13] using BWA v5.0.1 MEM module [51] with default parameters. SAMtools v1.12 software was used to convert mapping results into the BAM format and sort them. Duplicated reads were filtered with the Picard package v2.26.6 (https://broadinstitute.github.io/picard/). After BWA alignment, the reads around InDels were realigned, and realignment was performed with Genome Analysis Toolkit (GATK) v4.1.8.1 [52] in two steps. The first step used the RealignerTargetCreator package to identify regions where realignment was needed, and the second step used IndelRealigner to realign the regions found in the first step, which produced a realigned BAM file for each accession.

The variation detection followed the best practice workflow recommended by GATK. In brief, the variants were called for each accession by the GATK HaplotypeCaller. Sentieon v20211201 (https://www.sentieon.com) was used to perform joint genotyping on the gVCF files v0.1.17 [53] software, resulting in a comprehensive union of genetic variations. In the filtering step, (a) retain the variants with a minimum quality score of 30 (-minQ 30), (b) only keep sites with two alleles (-min-alleles 2 -max-alleles 2), (c) retain sites with at least four alleles carried by each site (-mac 4), (d) remove sites with a missing rate exceeding 15% the maximum allowed missing rate is 95% (-max-missing 0.95).

Sites with a minimum read depth of 3 were kept, and an error correction script was applied to further improve the data quality. The sex chromosome was not considered. InDels and SNPs with no bi-allelic, > 40% missing calls and MAF < 0.005 were removed, which yielded the basic set. SNPs with MAF < 0.05 were further removed for phylogenetic tree structure, IBS calculation, LD decay, PCA and population structure analyses.

SNPs and InDels annotation were performed according to the tea grey geometrid genome using the package ANNOVAR v2015-12–14 [54]. The coverage of each accession against each chromosome of the grapevine genome was counted based on an aligned BAM file using SAMtools v1.12 software. SNP density, InDel density, and total genetic diversity across each chromosome were counted with a 100 kb sliding window using VCFtools v0.1.17 [53] software.

### Phylogeny construction and population structure analysis

For all individuals, we first used PLINK v1.90b6.26 [55] with the parameters “-indep -pairphase 100 10 0.2” to determine a pruned SNP set to be used in the population structure analysis. In this way, we used 1,432,873 SNPs for the phylogeny and population analysis. The IBS genetic distance matrix was calculated to quantify the correlation between individuals using the “-distance 1-ibs flat-missing” parameter in PLINK. For the principal component analysis (PCA), we used PLINK with default parameters to extract the top 10 principal components (PCs). The top three PCs were plotted using package ggplot2 v3.4.2 [56] in R v4.2.0 (https://www.R-project.org). *E. obliqua* was used as the outgroup to root the phylogenetic tree. Maximum likelihood estimation of individual ancestries was performed using ADMIXTURE v1.3.0 [57] with the parameters “-cv -j4” for multiple repeats with different random seeds, and plotted in R. We used the web tool iTOLs (https://itol.embl.de/) to color the phylogenetic tree. We defined three phylogenetic groups according to the phylogenetic tree.

### Inference of population demographic history

For the demographic analysis, we used SMC +  + [58] v1.15.4. For SMC +  + , we selected more representative individuals from the three phylogenetic groups. *E. obliqua* was selected as the comparison species, and we used SNPable (http://lh3lh3.users.sourceforge.net/snpable.shtml), MSMC mappability, and BEDTools [59] v2.27.1 to prepare the input file for SMC +  + . In this analysis, the timepoints are specified as 1,000 ~ 1,000,000, the number of knots for the spline is 15, and the mutation rate is 2e-8.

### Genome scanning for selective sweep signals

We calculated the genetic diversity (*π*) using PLINK with the parameters “-window-pi 100,000”. We used PLINK to calculate the inter-population genetic differentiation index (*F*_ST_) of different phylogenetic groups in 100 kb windows [37]. We used PLINK to calculate Tajima’s *D* in 100-kb non-overlapping windows with parameter “-TajimaD 100000”.

We also performed a genetic differentiation and polymorphism levels (*θ*_*π*_, pairwise nucleotide variation as a measure of variability) based cross approach to investigate the selection signals across the whole genome. A 100 kb sliding window with a 10 kb step approach was applied to quantify *F*_ST_ and *θ*_*π*_ by using VCFtools v0.1.17 [53] software. The candidates that meet both top 5% of the two values were selected as selective signals.

### Migration events by TreeMix analysis

To infer migration events among the eight species, we used TreeMix v1.13 [60] to construct an ML tree with bighorn as the root using the “-noss” option to turn off the sample size correction, a window size (-K) of 500 SNPs (approximately 609 kb in this study) to account for the impact of LD, which is more than the average LD length of ~ 150 kb observed in sheep7. Blocks with 500 SNPs were resampled, and 100 bootstrap replications were performed. We constructed the ML trees with 0–11 migration events and corresponding residuals. The proportions of explained variance for the migration numbers were calculated using in-house scripts.

### Positive selection

To detect positive selection sites in *pgp-2* and *LCT*, a total of 9 *pgp-2* and 26 *LCT* were aligned using Multiple Alignment using Fast Fourier Transform (MAFFT) v7.480 software [61], respectively. This alignment helped to identify similarities and differences among the genes. Subsequently, dN/dS values were calculated using the site model in the CodeML tools of PAML v4.9j [62], employing the Bayes Empirical Bayes (BEB) method.

## Supplementary Information

**Additional file 1:** **Fig. S1.** The optimal K values from cross-validation (CV) error test for the analysis of Admixuture. **Fig. S2.** The optimum value of the migration edge (m) for TreeMix analysis. **Fig. S3.** The residual plot of TreeMix analysis. **Fig. S4. **The* π* value of SNPs in chromosome 15. **Fig. S5.** The Tajima’s*D* value of SNPs in chromosome 15.** Fig. S6.** GO enrichment of 222 candidate genes. **Fig. S7.** The phylogenetic tree of *pgp-2* genes. **Fig. S8.** The phylogenetic tree of* LCT*. **Fig. S9.** The phylogenetic tree of *COI* genes.

**Additional file 2:** **Table S1.** The site information of geometrid samples. **Table S2.** The statistic of CV error value. **Table S3**. The IBS matrix data. **Table S4.** The 20 vector quantities in PCA analysis. **Table S5.** The *π* value in 30 chromosomes. **Table S6.** The *F*_ST_value of 30 chromosomes. **Table S7.** The Tajima's *D* value of 30 chromosomes. **Table S8.** The function annotation and list of 222 candidate genes.** Table S9.** The Bayes Empirical Bayes (BEB) probabilities of all aligned sites in* pgp-2*. **Table S10.** The Bayes Empirical Bayes (BEB) probabilities of all aligned sites in *LCT* genes.

## Data Availability

Raw sequencing data that support the findings of this study can be found in the NCBI SRA database under the BioProject (PRJNA983079) with the accession numbers SRR25047583, SRR25047584, SRR25047585, SRR25047586, SRR25047587, SRR25047588, SRR25047589, SRR25047590, SRR25047591, SRR25047592, SRR25047593, SRR25047594, SRR25047595, SRR25047596, SRR25047597, SRR25047598, SRR25047599, SRR25047600, SRR25047601, SRR25047602, SRR25047603, SRR25047604, SRR25047605, SRR25047606, SRR25047607, SRR25047608, SRR25047609, SRR25047610, SRR25047611, SRR25047612, SRR25047613, SRR25047614, SRR25047615, SRR25047616, SRR25047617, SRR25047618, SRR25047619, SRR25047620, SRR25047621, SRR25047622, SRR25047623, SRR25047624, SRR25047625, SRR25047626, SRR25047627, SRR25047628, SRR25047629, SRR25047630, SRR25047631, SRR25047632, SRR25047633, SRR25047634, SRR25047635, SRR25047636. Additional data such as raw image files and in-house scripts that support this study are available from the first authors upon request.
