# Supplementary material for: Whole-genome resequencing of tea grey geometrid provides insights into their population structure and adaptation to tea crops
Source: Crop Health. 2024 Jun 1;2(1):8. doi: 10.1007/s44297-024-00026-z (PMC12825984; doi:10.1007/s44297-024-00026-z)
Supplement: Supplementary file 1 — Additional file 1: Fig. S1. The optimal K values from cross-validation (CV) error test for the analysis of Admixuture. Fig. S2. The optimum value of the migration edge (m) for TreeMix analysis. Fig. S3. The residual plot of TreeMix analysis. Fig. S4. The π value of SNPs in chromosome 15. Fig. S5. The Tajima’sD value of SNPs in chromosome 15. Fig. S6. GO enrichment of 222 candidate genes. Fig. S7. The phylogenetic tree of pgp-2 genes. Fig. S8. The phylogenetic tree of LCT. Fig. S9. The phylogenetic tree of COI genes. [file 44297_2024_26_MOESM1_ESM.docx]

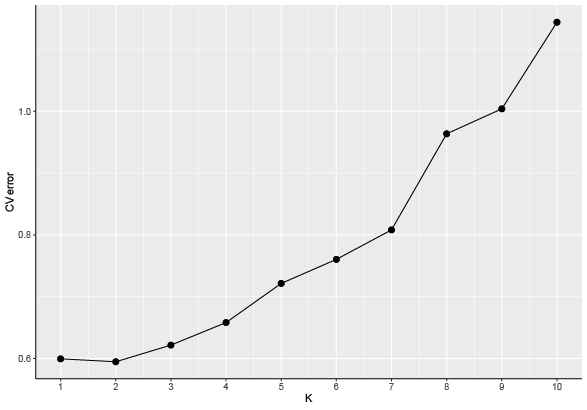


**Fig. S1. The optimal K values from cross-validation (CV) error test for the analysis of Admixuture.** The y axis represents the CV error, and the x axis represents potential K values.


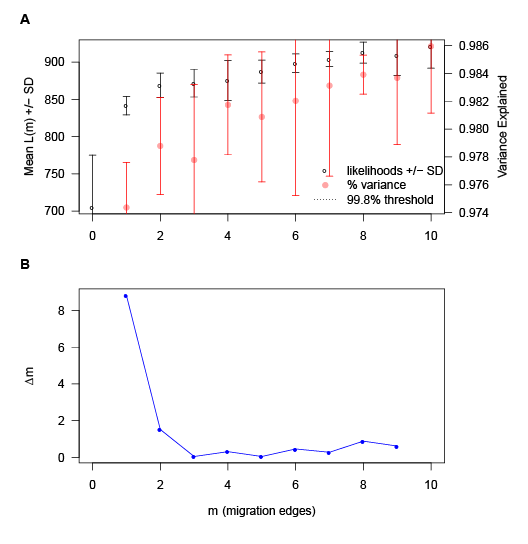


**Fig. S2. The optimum value of the migration edge (m) for TreeMix analysis. A** The y axis on the left represents mean L (m) +/- SD (Standard Deviation) for each potential m values, and the y axis on the right represents variance explained. The x axis represents m values. **B** The △ (delta) m for each potential m values. The x axis represents the statistic of delta m, and the y axis represents m values.


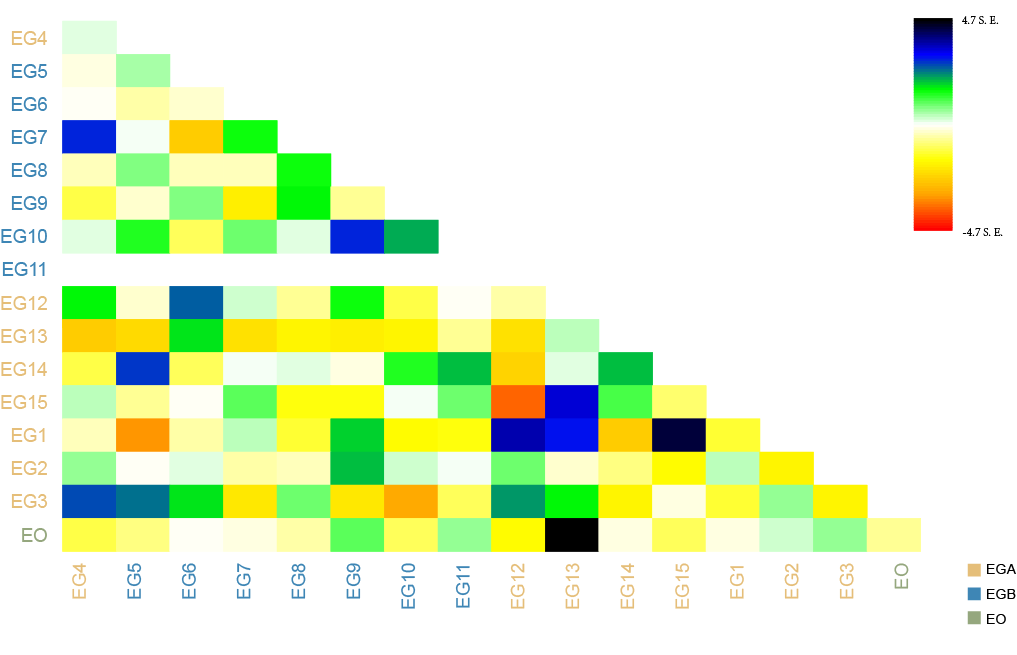


**Fig. S3.** **The residual plot of TreeMix analysis.** The color range of rectangles means standard error (S. E.) value of migration weight. Sample labels of EGA were colored in “slight orange”, EGB were colored in “blue” and EO was colored in “green”.


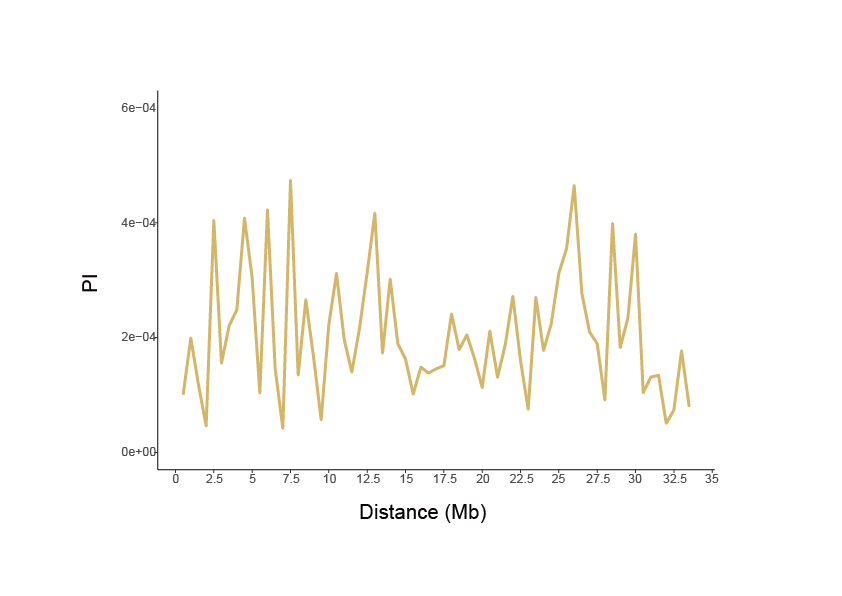


**Fig. S4. The *π* value of SNPs in chromosome 15.** The y axis represents the statistic of *π* values, and the x axis represents the distance of chromosome 15 (Mb).


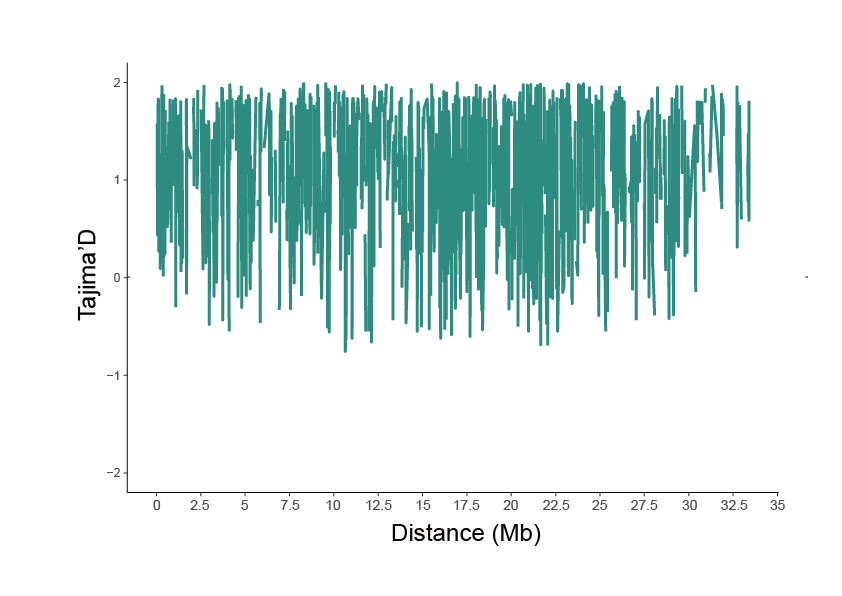


**Fig. S5. The Tajima’ *D* value of SNPs in chromosome 15.** The y axis represents the statistic of *π* values, and the x axis represents the distance of chromosome 15 (Mb).


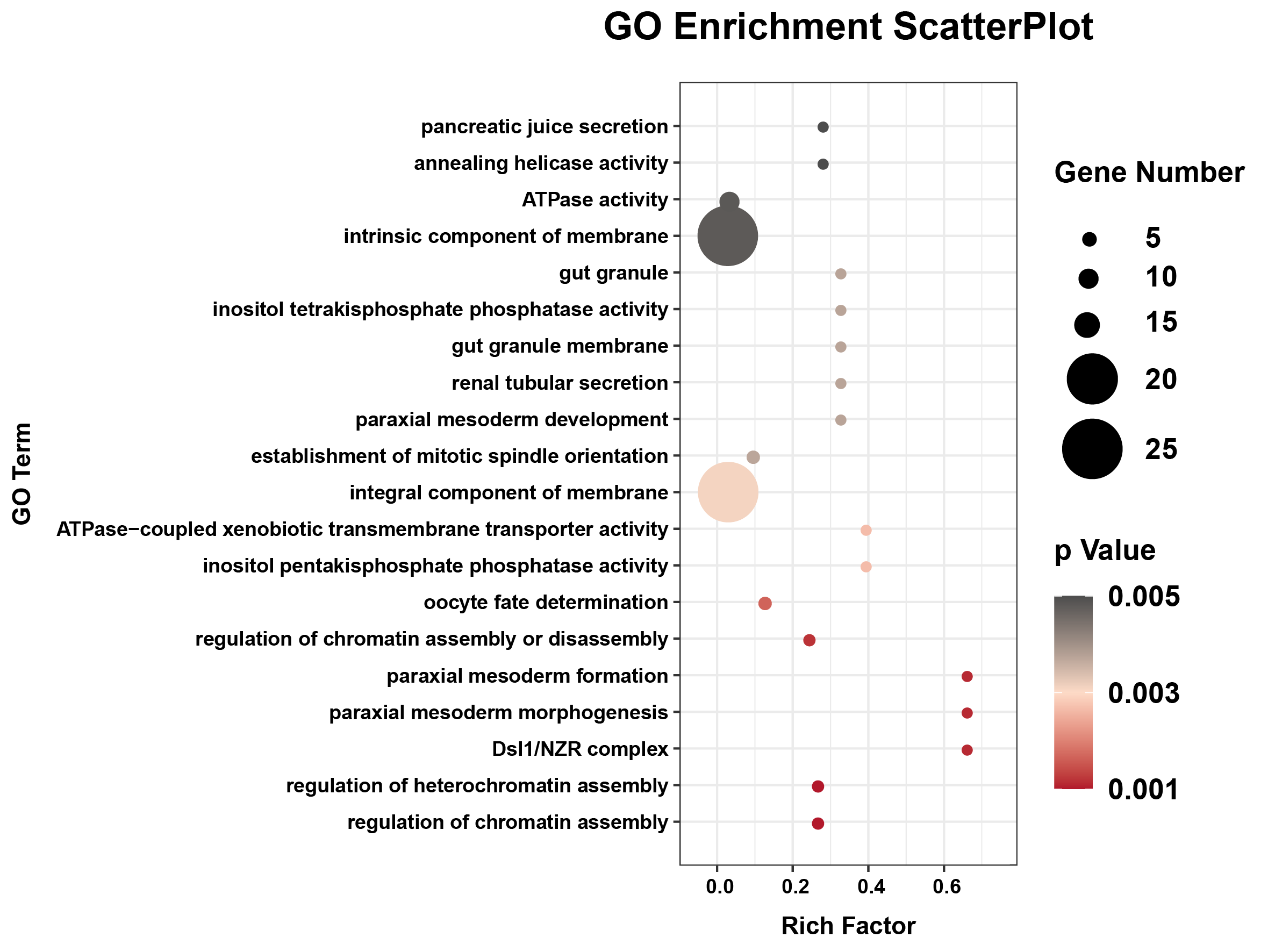


**Fig. S6. GO enrichment of 222 candidate genes.** The y axis represents the GO term, and the x axis represents the rich factor.


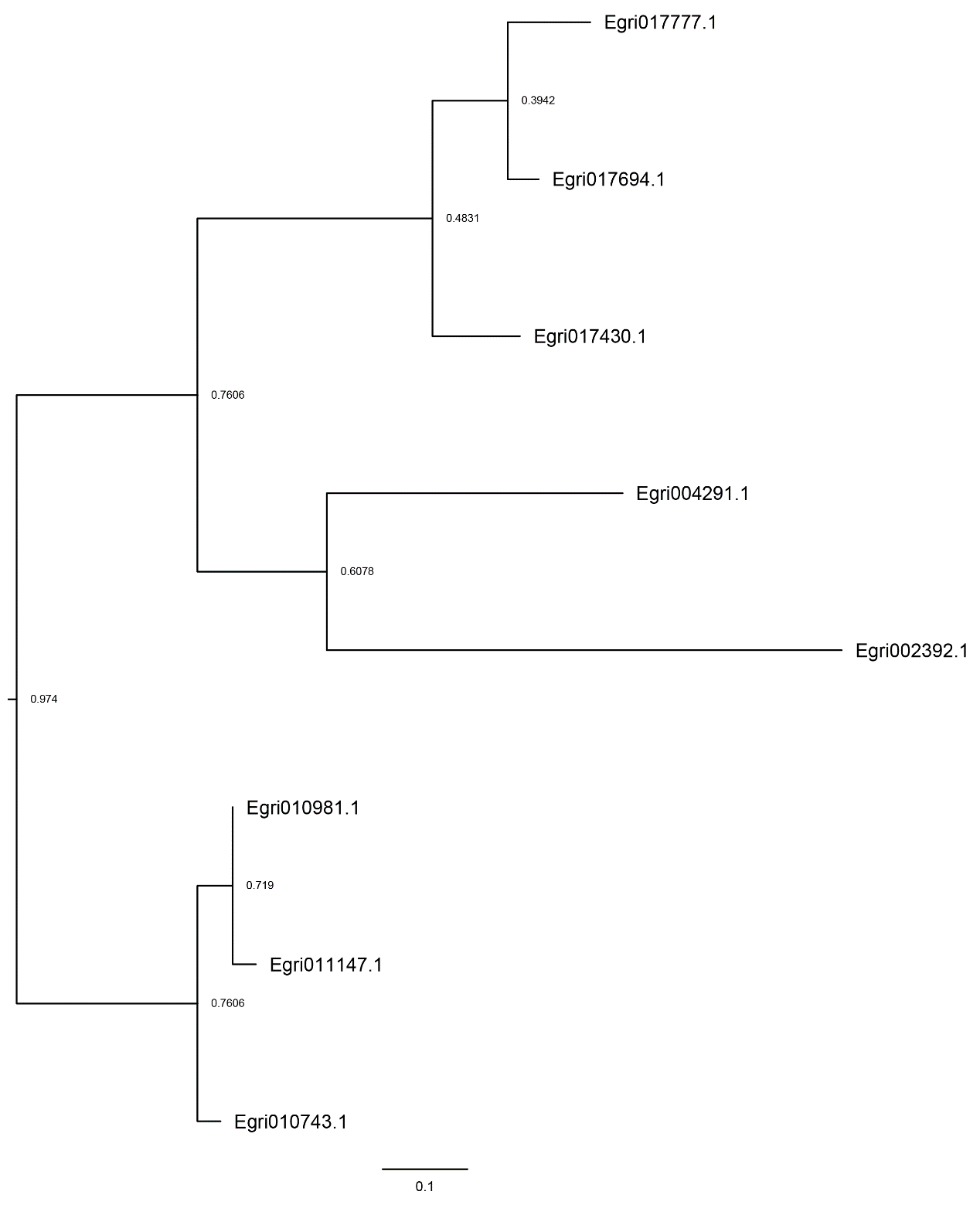


**Fig. S7. The phylogenetic tree of 9 *pgp-2* genes.** The phylogenetic was constructed by the maximum likelihood (ML) method, and the bootstrap values are indicated on the node.


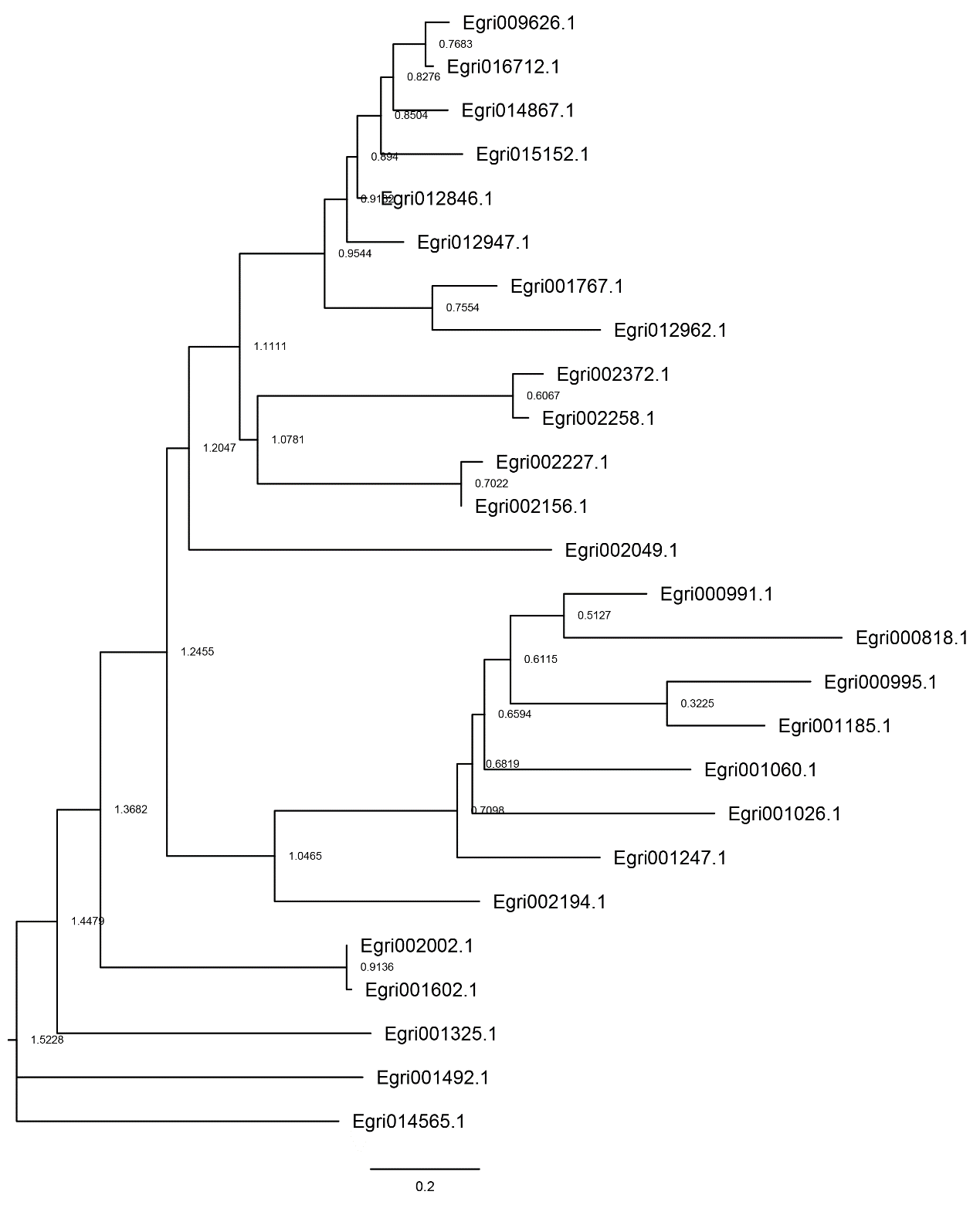


**Fig. S8. The phylogenetic tree of 26 *LCT* genes.** The phylogenetic was constructed by the maximum likelihood (ML) method, and the bootstrap values are indicated on the node.


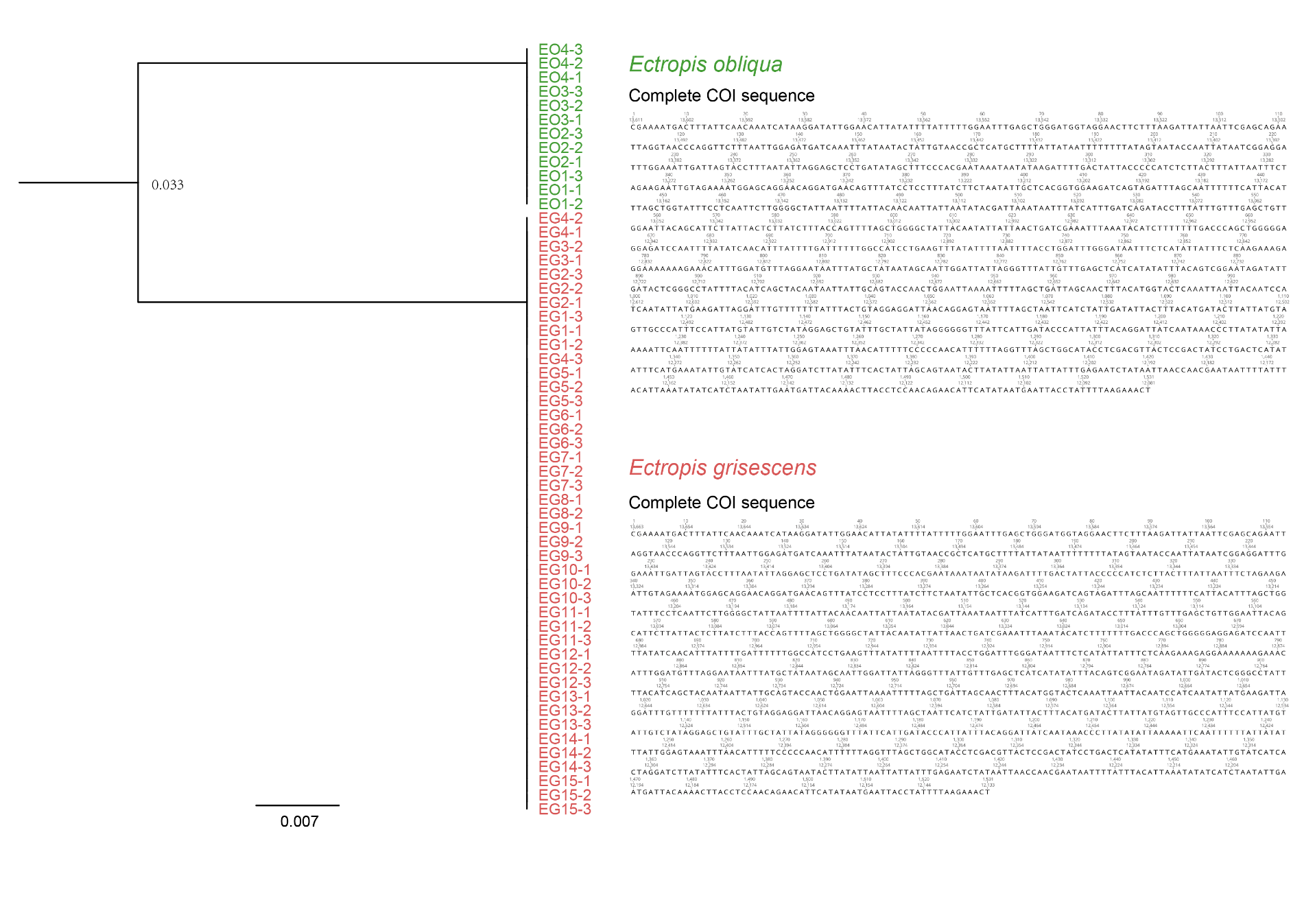


**Fig. S9. The phylogenetic tree of *COI* genes.** The phylogenetic was constructed by the Neighbor-Joining (NJ) method, and the bootstrap values are indicated on the node. Complete COI sequences are exhibited.
